# Outcomes of Veterans Treated in Veterans Affairs Hospitals vs Non–Veterans Affairs Hospitals

**DOI:** 10.1001/jamanetworkopen.2023.45898

**Published:** 2023-12-01

**Authors:** Jean Yoon, Ciaran S. Phibbs, Michael K. Ong, Megan E. Vanneman, Adam Chow, Andrew Redd, Kenneth W. Kizer, Matthew P. Dizon, Emily Wong, Yue Zhang

**Affiliations:** 1 Health Economics Resource Center, Veterans Affairs Palo Alto Health Care System, Menlo Park, California; 2 Center for Innovation to Implementation, Veterans Affairs Palo Alto Health Care System, Menlo Park, California; 3 Department of General Internal Medicine, University of California San Francisco School of Medicine, San Francisco; 4 Departments of Pediatrics and Health Policy, Stanford University School of Medicine, Stanford, California; 5 Veterans Affairs Center for the Study of Healthcare Innovation, Implementation and Policy, Los Angeles, California; 6 Department of Health Policy and Management, Fielding School of Public Health, University of California, Los Angeles; 7 Department of Medicine, David Geffen School of Medicine, University of California, Los Angeles; 8 Informatics, Decision-Enhancement and Analytic Sciences Center, Veterans Affairs Salt Lake City Health Care System, Salt Lake City, Utah; 9 Division of Epidemiology, Department of Internal Medicine, University of Utah School of Medicine, Salt Lake City; 10 Division of Health System Innovation and Research, Department of Population Health Sciences, University of Utah School of Medicine, Salt Lake City; 11 Division of Biostatistics, Department of Population Health Sciences, University of Utah School of Medicine, Salt Lake City; 12 Stanford University School of Medicine, Stanford, California

## Abstract

**Importance:**

Many veterans enrolled in the Veterans Affairs (VA) health care system have access to non-VA care through insurance and VA-purchased community care. Prior comparisons of VA and non-VA hospital outcomes have been limited to subpopulations.

**Objective:**

To compare outcomes for 6 acute conditions in VA and non-VA hospitals for younger and older veterans using VA and all-payer discharge data.

**Design, Setting, and Participants:**

This cohort study used a repeated cross-sectional analysis of hospitalization records for acute myocardial infarction (AMI), coronary artery bypass graft (CABG), gastrointestinal (GI) hemorrhage, heart failure (HF), pneumonia, and stroke. Participants included VA enrollees from 11 states at VA and non-VA hospitals from 2012 to 2017. Analysis was conducted from July 1, 2022, to October 18, 2023.

**Exposures:**

Treatment in VA or non-VA hospital.

**Main Outcome and Measures:**

Thirty-day mortality, 30-day readmission, length of stay (LOS), and costs. Average treatment outcomes of VA hospitals were estimated using inverse probability weighted regression adjustment to account for selection into hospitals. Models were stratified by veterans’ age (aged less than 65 years and aged 65 years and older).

**Results:**

There was a total of 593 578 hospitalizations and 414 861 patients with mean (SD) age 75 (12) years, 405 602 males (98%), 442 297 hospitalizations of non-Hispanic White individuals (75%) and 73 155 hospitalizations of non-Hispanic Black individuals (12%) overall. VA hospitalizations had a lower probability of 30-day mortality for HF (age ≥65 years, −0.02 [95% CI, −0.03 to −0.01]) and stroke (age <65 years, −0.03 [95% CI, −0.05 to −0.02]; age ≥65 years, −0.05 [95% CI, −0.07 to −0.03]). VA hospitalizations had a lower probability of 30-day readmission for CABG (age <65 years, −0.04 [95% CI, −0.06 to −0.01]; age ≥65 years, −0.05 [95% CI, −0.07 to −0.02]), GI hemorrhage (age <65 years, −0.04 [95% CI, −0.06 to −0.03]), HF (age <65 years, −0.05 [95% CI, −0.07 to −0.03]), pneumonia (age <65 years, −0.04 [95% CI, −0.06 to −0.03]; age ≥65 years, −0.03 [95% CI, −0.04 to −0.02]), and stroke (age <65 years, −0.11 [95% CI, −0.13 to −0.09]; age ≥65 years, −0.13 [95% CI, −0.16 to −0.10]) but higher probability of readmission for AMI (age <65 years, 0.04 [95% CI, 0.01 to 0.06]). VA hospitalizations had a longer mean LOS and higher costs for all conditions, except AMI and stroke in younger patients.

**Conclusions and Relevance:**

In this cohort study of veterans, VA hospitalizations had lower mortality for HF and stroke and lower readmissions, longer LOS, and higher costs for most conditions compared with non-VA hospitalizations with differences by condition and age group. There were tradeoffs between better outcomes and higher resource use in VA hospitals for some conditions.

## Key Points

**Question:**

How do outcomes compare in Veterans Affairs (VA) hospitals and non-VA hospitals for 6 conditions for veterans aged less than 65 years and veterans 65 years and older?

**Findings:**

In this cohort study of 593 578 hospitalizations and 414 861 patients, VA hospitalizations compared with non-VA hospitalizations had significantly lower 30-day mortality for heart failure and stroke, lower 30-day readmission for coronary artery bypass graft, gastrointestinal hemorrhage, heart failure, pneumonia, and stroke, but longer mean length of stay and higher mean costs for most conditions. There were differences by age group.

**Meaning:**

These findings suggest that veterans had better outcomes in VA hospitals for some conditions at the expense of higher costs.

## Teaser

This cohort study compares outcomes for 6 acute conditions in Veterans Affairs (VA) and non-VA hospitals in 11 states for veterans using VA and all-payer discharge data.

## Introduction

The Veterans Affairs (VA) health care system is the only national integrated delivery system in the US. Many of the 9 million veterans enrolled in the VA have access to non-VA care through VA-purchased services from community clinicians or concomitant enrollment in insurance programs. The VA has long purchased community care when services could not be provided on site, but the Veterans Access, Choice and Accountability Act (Choice Act) in 2014 followed by the VA Maintaining Internal Systems and Strengthening Integrated Outside Networks (MISSION) Act in 2018 expanded the criteria to purchase care for veterans experiencing access barriers.^1,2^ The Patient Protection and Affordable Care Act further expanded access to Medicaid for low-income adults, including veterans, in many states beginning in 2014.^1^ These policies increased use of non-VA care and decreased use of VA services.^2,3^

Increased access to non-VA care can lead to better outcomes if patients receive higher-quality or more timely care.^4^ However, studies comparing quality of VA and private clinicians documented process and outcome measures for VA care that were equivalent to or superior to non-VA care for surgical procedures, some hospital care, and preventive care.^5,6,7,8^ Many of these prior studies were limited to older veterans using VA services and older patients using Medicare services, including many nonveterans, due to wide availability of Medicare data.^8,9,10,11,12,13,14^ However, the veteran enrollee population is more male and has worse health status, greater disability, and lower incomes compared with the nonveteran population.^15,16^ Moreover, younger veterans are typically not included in comparisons due to a lack of comprehensive data on non-VA use outside of Medicare, which reduces the generalizability of these comparisons. Other studies compared VA and community care purchased by the VA and focused on select subpopulations having a particular condition or receiving a particular procedure.^17,18,19,20,21^

Inpatient care is a core service provided by the VA in 140 hospitals with medical or surgical acute care beds, which range widely in volume and service capabilities. Veterans are like other patients insofar as distance to clinicians and travel time influence their preferred choice of clinicians, especially for inpatient care.^22,23,24^ At a time when veterans have more access to non-VA hospital care, it is important to examine differences in outcomes between VA and non-VA hospitals.

This study compared mortality, readmission, length of stay (LOS), and costs of veterans hospitalized in VA and non-VA hospitals for acute myocardial infarction (AMI), coronary artery bypass surgery (CABG), gastrointestinal (GI) hemorrhage, heart failure (HF), pneumonia, and stroke. A lack of data on non-VA utilization often hinders comparisons between VA and non-VA care, but we used a comprehensive data set of VA and non-VA all-payer inpatient care records. No studies to date compared hospital outcomes for veterans of all ages with access to VA care.

## Methods

The cohort study was approved by the institutional review boards (IRBs) at Stanford University, University of Utah, and Greater Los Angeles VA with a waiver of consent granted by the IRBs. We followed the Strengthening the Reporting of Observational Studies in Epidemiology (STROBE) reporting guideline for reporting cohort studies.

### Study Cohort and Data Sources

We conducted a study using repeated cross-sections of hospitalizations for VA enrollees discharged January 1, 2012 to December 31, 2017. After reviewing availability and policies to request all-payer discharge data for research in all states, we obtained hospitalization records in 11 geographically diverse states (ie, Arizona, California, Connecticut, Florida, Illinois, Louisiana, Massachusetts, Missouri, New York, Pennsylvania, and South Carolina), which allowed linkage between discharge data and VA enrollment data. Our sample of states represented the Northeastern, Southeastern, Midwestern, and Western regions of the US; approximately 38% of VA enrollees live in these states.^25^

Veterans’ VA use and cost records were obtained from the Inpatient Encounter files and the Managerial Cost Accounting (MCA) files in the VA Informatics and Computing Infrastructure.^26^ Veterans’ non-VA use records were obtained from state inpatient discharge data linked with VA enrollment data using either deterministic or probabilistic methods with personal identifiers.

We obtained patients’ sociodemographic characteristics from the VA Health Enrollment Files and the VA Observational Medical Outcomes Partnership Files.^27,28^ Veterans’ and VA hospitals’ addresses were obtained from the VA Geospatial Services Support Center Files.^29^ Non-VA hospitals’ addresses were obtained from the Centers for Medicare & Medicaid Services (CMS) Provider of Service File.^30^ Veterans’ death information was obtained from the VA Vital Status File. VA hospital characteristics were obtained from the Veterans Integrated Service Network Support Services Center, and non-VA hospital characteristics were obtained from CMS hospital cost reports.^31,32^

### Acute Medical or Surgical Hospital Stays

VA acute hospital stays were identified from medicine and surgery bed sections and diagnosis-related group (DRG). We excluded stays within 30 days of discharge from a previous admission and stays for more than 180 days (not considered acute). Hospitalizations for AMI, CABG, GI hemorrhage, HF, pneumonia, and stroke were identified from principal diagnosis codes. We focused on these conditions since the Agency for Healthcare research and Quality uses hospital mortality for these conditions as a quality indicator.^33^ Discharge records for Illinois could not be obtained for 2012, so hospitalizations in Illinois were excluded in that year. The Figure shows how the final sample was derived.

**Figure.**
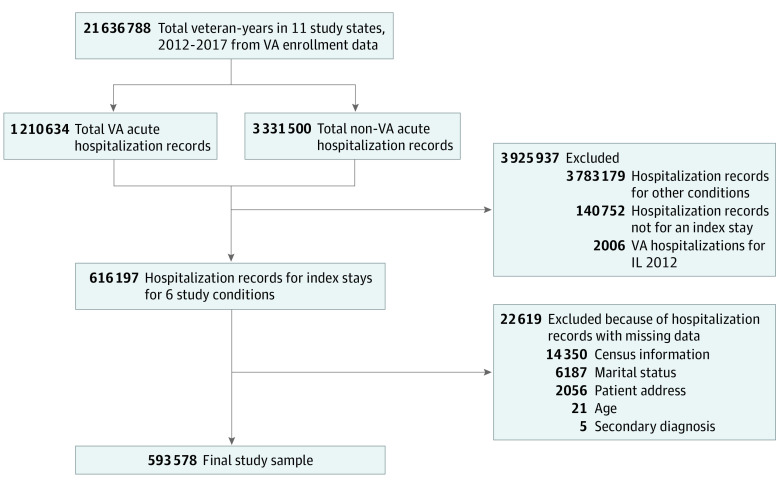
Study Sample Flowchart Study sample inclusion and exclusion criteria. VA indicates Veterans Affairs.

### Outcome Measures

Hospital outcomes included 30-day hospital mortality, 30-day readmission, inpatient costs, and LOS. Hospital mortality was indicated for all-cause deaths occurring within 30 days of admission. Thirty-day all-cause readmission was indicated for stays followed by another admission within 30 days of discharge regardless of where the stays occurred. Thirty-day mortality could not be measured for non-VA hospital stays in California and Pennsylvania since admission and discharge dates were not provided, and 30-day readmissions could not be measured for non-VA stays in California because no readmission indicator was provided in the discharge data.

VA costs included direct and indirect costs after subtracting national administration costs.^34^ Non-VA costs included estimated professional fees^35^ and facility charges which were adjusted by hospital cost-to-charge ratios.^31^ Costs were adjusted for inflation to 2017 dollars.^36,37^ LOS was calculated as the number of days between admission and discharge, inclusive.

### Statistical Analysis

The unit of analysis was the hospital stay. Since patients who were more sick may potentially choose 1 hospital system over another, comparing outcomes in a traditional regression may produce biased results. Therefore, we used doubly robust methods with inverse probability weighted regression adjustment (IPWRA).^38,39,40^ In IPWRA models, we estimate 1 equation for treatment (in a VA hospital) and another for outcomes. Observations are weighted by the inverse of their conditional probability of treatment (admitted to a VA hospital) in a regression estimating outcomes so that patients are balanced in their covariates (eMethods in Supplement 1 and eTables 13-18 and eTable 22 in Supplement 2). The advantage of this method is that only 1 of the treatment and outcome equations needs to be correctly specified to produce unbiased results. Outcomes were estimated for each condition and age group separately. Analysis was conducted in StataMP version 18 (StataCorp) using teffects and took place from July 1, 2022, to October 18, 2023.

#### Patient Measures in Treatment Equation

We estimated treatment in VA vs non-VA hospital in a probit model by adjusting for patient factors influencing use of VA hospitals,^8,41,42,43^ including patients’ age, sex, race and ethnicity (measured in electronic health record), marital status, priority for VA care, distance to nearest VA hospital, comorbidity score, comorbidity for substance use disorders and posttraumatic stress disorder, geographic region (Northeast, South, Midwest, West), rural or urban location, area-level income (mean standardized), and post-Choice Act period. Race and ethnicity were included to adjust for sociodemographic factors. Comorbidity score was measured for each stay using the Elixhauser-van Walvraven index from all recorded diagnosis codes.^44^ We indicated post-Choice Act period beginning in 2015, the first full year of implementation, because it reduced VA use. Median income was obtained for patients’ zip code from US Census data.

#### Patient Measures in Outcomes Equation

In outcomes equations, we adjusted for factors potentially influencing outcomes that included patients’ age, marital status, priority for VA care, nonelective admission, overall comorbidity score, specific medical comorbidities, mental health comorbidity, and area-level income. Models for mortality and readmission used a probit model, and models for LOS and log-transformed costs used a linear model. We estimated average treatment outcomes of VA hospitals as the difference between estimated probabilities and means for all observations assuming treatment in VA hospitals and all observations assuming treatment in non-VA hospitals along with 95% CIs. Standard errors were adjusted for each unique patient-hospital combination.^45^

In sensitivity analyses, we estimated in-hospital mortality because we had complete data for all states. We also conducted analysis limited to nonelective hospitalizations because treatment and outcome patterns may vary by admission type and analysis with only 1 observation per patient throughout the 6-year period.

For descriptive purposes, hospital characteristics were measured in VA and non-VA hospitals, including number of staffed beds, bed occupancy rate, academic affiliation, and patient experience rating using percent of patients likely to recommend their hospital. Patient and hospital characteristics by VA and non-VA hospital and age group were compared in Pearson χ^2^ and analysis of variance tests.

## Results

### Characteristics of Patients and Hospitals by System

The study sample included a total of 593 578 hospitalizations and 414 861 veterans with a mean (SD) age 75 (12) years, 405 602 males (98%), 73 155 hospitalizations of non-Hispanic Black individuals (12%), and 442 297 hospitalizations of non-Hispanic White individuals (75%) overall. The mean age was similar for younger veterans but higher for older veterans in non-VA hospitalizations compared with VA hospitalizations (Table 1). Most patients were male in all groups. Non-VA hospitalizations had higher mean comorbidity scores. VA hospitalizations were more likely to be for patients who were Black individuals or Hispanic individuals, not currently married, had a service-connected disability, and lived in urban areas and closer to a VA hospital than non-VA hospitalizations. Patients traveled farther when admitted to a VA hospital vs non-VA hospital.

**Table 1.** Unweighted Patient and Hospital Characteristics of VA and Non-VA Hospitalizations, 2012-2017^a^

Patient characteristics	Patients age <65 years, No. (%)		*P* value^b^	Patients age ≥65 years, No. (%)		*P* value^b^
	VA (n = 30 372)	Non-VA (n = 75 440)		VA (n = 70 266)	Non-VA (n = 417 500)	
Age, mean (SD), y	57 (7)	57 (7)	.58	77 (9)	80 (9)	<.001
Sex						
Male	28 968 (95.4)	71 922 (95.3)	.78	69 119 (98.4)	411 091 (98.5)	.05
Female	1404 (4.6)	3518 (4.7)		1147 (1.6)	6409 (1.5)	
Elixhauser-van Walraven Comorbidity Score, mean (SD)	4.6 (6.8)	5.7 (7.5)	<.001	7.4 (6.5)	9.1 (7.3)	<.001
Race and ethnicity						
Non-Hispanic Black	9630 (31.7)	19 887 (26.4)	<.001	12 136 (17.3)	31 502 (7.6)	<.001
Hispanic	2288 (7.5)	4764 (6.3)		4062 (5.8)	14 792 (3.5)	
Non-Hispanic White	16 374 (53.9)	45 216 (59.9)		48 775 (69.4)	331 932 (79.5)	
Other^c^	955 (3.1)	2612 (3.5)		2054 (2.9)	10 912 (2.6)	
Unknown	1125 (3.7)	2961 (3.9)		3239 (4.6)	28 362 (6.8)	
Marital status						
Currently married	10 246 (33.7)	32 944 (43.7)	<.001	32 183 (45.8)	270 574 (64.8)	<.001
Divorced, widowed, or separated	13 860 (45.6)	29 897 (39.6)		32 081 (45.7)	128 406 (30.8)	
Single never married	6266 (20.6)	12 599 (16.7)		6002 (8.5)	18 520 (4.4)	
VA enrollment priority group						
Service-connected disability						
>30%	9732 (32.0)	21 872 (29.0)	<.001	24 788 (35.3)	87 652 (21.0)	<.001
10%-20%	5340 (17.6)	13 334 (17.7)		13 045 (18.6)	69 493 (16.7)	
Below means test, 5 y postdischarge	12 176 (40.1)	27 888 (37.0)		24 145 (34.4)	95 002 (22.8)	
Above means test	3119 (10.3)	12 343 (16.4)		8287 (11.8)	165 340 (39.6)	
Rurality						
Urban	24 828 (81.8)	55 510 (73.6)	<.001	54 805 (78.0)	301 386 (72.2)	<.001
Rural	5544 (18.2)	19 930 (26.4)		15 461 (22.0)	116 114 (27.8)	
Distance to closest VA hospital, in miles, mean (SD)	24 (27)	44 (39)	<.001	24 (25)	43 (37)	<.001
Distance to admitted hospital, in miles, mean (SD)	26 (28)	15 (32)	<.001	26 (28)	12 (25)	<.001
Area median household income, mean (SD), $	51 557 (20 398)	52 581 (19 034)	<.001	55 954 (23 037)	59 953 (23 365)	<.001
Area unemployment rate, mean (SD)	6.2% (2.8)	6.2% (2.9)	.17	6.0% (2.7)	5.9% (2.7)	.002
Payer of non-VA care						
Medicare	NA	23 755 (31.5)	NA	NA	372 600 (89.3)	NA
VA-purchased	NA	12 485 (16.6)		NA	12 737 (3.1)	
Private	NA	17 147 (22.7)		NA	19 432 (4.7)	
Medicaid	NA	8430 (11.2)		NA	1144 (0.3)	
Other	NA	13 623 (18.1)		NA	11 587 (2.8)	
Nonelective admission	28 283 (93.1)	68 421 (90.7)	<.001	66 284 (94.3)	373 761 (89.5)	<.001
Admitting condition						
AMI	3847 (12.7)	19 085 (25.3)	<.001	7418 (10.6)	67 395 (16.1)	<.001
CABG	1640 (5.4)	4256 (5.6)	.12	2548 (3.6)	15 981 (3.8)	.01
GI hemorrhage	4908 (16.2)	10 313 (13.7)	<.001	9385 (13.4)	56 334 (13.5)	.33
Heart failure	9625 (31.7)	17 472 (23.2)	<.001	27 306 (38.9)	131 084 (31.4)	<.001
Pneumonia	7065 (23.3)	12 684 (16.8)	<.001	17 290 (24.6)	89 024 (21.3)	<.001
Stroke	3412 (11.2)	13 070 (17.3)	<.001	6493 (9.2)	62 029 (14.9)	<.001
Medical comorbidity						
Heart failure	1553 (5.1)	6705 (8.9)	<.001	7057 (10.0)	66 194 (15.9)	<.001
Valvular disease	382 (1.3)	1919 (2.5)	<.001	2261(3.2)	29 576 (7.1)	<.001
Peripheral vascular disease	1694 (5.6)	6125 (8.1)	<.001	7180 (10.2)	59 964 (14.4)	<.001
Cardiac arrhythmias	6604 (21.7)	18 568 (24.6)	<.001	28 884 (41.1)	198 361 (47.5)	<.001
Neurological disorders	1189 (3.9)	4063 (5.4)	<.001	5158 (7.3)	37 853 (9.1)	<.001
COPD	7901 (26.0)	21 650 (28.7)	<.001	23 534 (33.5)	137 508 (32.9)	.004
Diabetes without chronic complications	9192 (30.3)	19 995 (26.5)	<.001	22 314 (31.8)	107 743 (25.8)	<.001
Diabetes without chronic complications	3176 (10.5)	10 091 (13.4)	<.001	8042 (11.5)	54 778 (13.1)	<.001
Hypothyroidism	1794 (5.9)	4633 (6.1)	.15	7375 (10.5)	58 511 (14.0)	<.001
Kidney failure	5944 (19.6)	14 960 (19.8)	.34	23 283 (33.1)	145 971 (35.0)	<.001
Liver disease	3772 (12.4)	6090 (8.1)	<.001	3214 (4.6)	10 460 (2.5)	<.001
Lymphoma	298 (1.0)	599 (0.8)	.003	1023 (1.5)	5346 (1.3)	<.001
Metastatic cancer	452 (1.5)	1014 (1.3)	.07	1457 (2.1)	7732 (1.9)	<.001
Solid tumor without metastasis	926 (3.1)	1396 (1.9)	<.001	4071 (5.8)	14 001(3.4)	<.001
Rheumatoid arthritis	474 (1.6)	1279 (1.7)	.12	1148 (1.6)	8891 (2.1)	<.001
Coagulopathy	1291 (4.3)	5242 (7.0)	<.001	3187 (4.5)	34 284 (8.2)	<.001
Obesity	3900 (12.8)	15 189 (20.1)	<.001	5299 (7.5)	42 717 (10.2)	<.001
Weight loss	642 (2.1)	2919 (3.9)	<.001	1828 (2.6)	20 829 (5.0)	<.001
Fluid and electrolyte disorders	5015 (16.5)	19 891 (26.4)	<.001	12 096 (17.2)	115 259 (27.6)	<.001
Chronic blood loss anemia	494 (1.6)	1295 (1.7)	.30	1216 (1.7)	8520 (2.0)	<.001
Deficiency anemias	4985 (16.4)	12 202 (16.2)	.34	15 543 (22.1)	97 414 (23.3)	<.001
Mental health comorbidity						
Mood disorders	5977 (19.7)	12 297 (16.3)	<.001	8619 (12.3)	41 625 (10.0)	<.001
Serious mental illness	1989 (6.6)	3928 (5.2)	<.001	1792 (2.6)	6283 (1.5)	<.001
Substance use disorders	5606 (18.5)	13 036 (17.3)	<.001	3619 (5.2)	14 483 (3.5)	<.001
Posttraumatic stress disorder	2407 (7.9)	3622 (4.8)	<.001	3845 (5.5)	5894 (1.4)	<.001
Hospital characteristics, No.	45	1446	NA	45	1552	NA
Total beds, mean (SD)	124 (57)	214 (219)	.007	125 (58)	203 (215)	.02
Occupancy rate, mean (SD)	0.66 (0.18)	0.54 (0.19)	<.001	0.65 (0.17)	0.54 (0.20)	<.001
Academic affiliation, mean (SD)	0.58 (0.50)	0.36 (0.48)	.003	0.64 (0.48)	0.34 (0.47)	<.001
Patient experience, mean (SD)	63.6 (10.9)	69.1 (9.5)	<.001	63.5 (10.5)	69.3 (9.6)	<.001

Abbreviations: AMI, acute myocardial infarction; CABG, coronary artery bypass surgery; COPD, chronic obstructive pulmonary disease; GI, gastrointestinal; NA, not applicable; VA, Veterans Affairs

^a^ Observations summarized here are hospitalizations.

^b^ *P* values reported for Pearson χ^2^ tests for categorical variables and analysis of variance tests for continuous variables.

^c^ Includes Alaska Native, Asian American, Native Hawaiian, and Pacific Islander.

VA hospitalizations were more likely to be nonelective and for HF and pneumonia compared with other study conditions than non-VA hospitalizations. Rates of medical comorbidities were generally lower among VA hospitals compared with non-VA hospitals (Table 1).

The mean (SD) number of hospital beds was lower in VA hospitals compared with non-VA hospitals (age <65 years, 124 [57] vs 214 [219]; *P* = .007; age ≥65 years, 125 [58] vs 203 [215]; *P* = .02), and the mean (SD) hospital bed occupancy rate was higher in VA hospitals than non-VA hospitals (age <65 years, 0.66 [0.18] vs 0.54 [0.19]; *P* <.001; age ≥65 years, 0.65 [0.17] vs 0.54 [0.20]; *P* <.001). A higher proportion of VA hospitals had a major academic affiliation (mean [SD] age <65 years, 0.58 [0.50] vs 0.36 [0.48]; *P* = .003; age ≥65 years, 0.64 [0.48] vs 0.34 [0.47]; *P* <.001), and mean (SD) patient experience rating was lower for VA hospitals (age <65 years, 63.6 [10.9] vs 69.1 [9.5]; *P* <.001; age ≥65 years, 63.5 [10.5] vs 69.3 [9.6]; *P* <.001).

### Unweighted Hospital Outcomes

VA hospitalizations compared with non-VA hospitalizations had lower unweighted probability of 30-day mortality among older patients for AMI (age ≥65 years, 548 of 5601 [9.8%] vs 5106 of 42 715 [12.0%]; *P* <.001), GI hemorrhage (288 of 6987 [4.1%] vs 2119 of 36 482 [5.8%]; *P* <.001), HF (1235 of 20 648 [6.0%] vs 8742 of 84 465 [10.4%]; *P* <.001), pneumonia (965 of 13 417 [7.2%] vs 5785 of of 59 555 [9.7%]; *P* <.001), and stroke (331 of 4726 [7.0%] vs 6494 of 39 266 [16.5%]; *P* <.001) (Table 2). VA hospitalizations compared with non-VA hospitalizations had lower unweighted probability of 30-day readmission for both age groups for CABG (age <65 years, 170 of 1637 [10.4%] vs 486 of 3389 [14.3]; *P* < .001; age ≥65 years, 355 of 2537 [14.0%] vs 2627 of 12 835 [20.5%]; *P* < .001), GI hemorrage (age <65 years, 700 of 4871 [14.4%] vs 1466 of 7663 [19.1%]; *P* < .001; age ≥65 years, 1608 of 9287 [17.3%] vs 8275 of 44 675 [18.5%]; *P* = .006), HF (age <65 years, 1993 of 9523 [20.9%] vs 3322 of 12 498 [26.6%]; *P* < .001; age ≥65 years, 6009 of 26 980 [22.3%] vs 25 672 of 104 445 [24.6%]; *P* < .001), pneumonia (age <65 years, 1083 of 7022 [15.4%] vs 1894 of 10 029 [18.9%]; *P* < .001; age ≥65 years, 2817 of 17 090 [16.5%] vs 13 934 of 72 321[19.3l%]; *P* < .001), and stroke (age <65 years, 465 of 3389 [13.7%] vs 2764 of 10 065 [27.5%]; *P* < .001; age ≥65 years, 1000 of 6445 [15.5%] vs 14 512 of 48 456 [30.0%]; *P* < .001).

**Table 2.** Unweighted Acute Hospitalization Outcomes in VA and Non-VA Hospitals, 2012-2017

Condition by age group, y^a^	No.	30-d mortality		*P* value^b^	30-d readmission		*P* value	LOS, d		*P* value	Cost (in $1000s)		*P* value
		Unweighted, No. (%)			Unweighted, No. (%)			Unweighted, mean (SD)			Unweighted, mean (SD)		
		VA	Non-VA		VA	Non-VA		VA	Non-VA		VA	Non-VA	
**AMI**													
<65	22 681	83 (2.9)	468 (3.7)	.04	791 (20.7)	2795 (18.7)	.004	4.1 (6.2)	4.0 (4.6)	.15	22.5 (35.4)	23.8 (24.9)	.005
≥65	74 138	548 (9.8)	5106 (12.0)	<.001	1777 (24.2)	12 922 (24.5)	.54	5.2 (6.4)	4.7 (5.0)	<.001	24.6 (32.0)	21.7 (24.8)	<.001
**CABG**													
<65	5829	12 (1.0)	39 (1.4)	.35	170 (10.4)	486 (14.3)	<.001	10.5 (8.9)	8.9 (5.8)	<.001	68.5 (52.8)	51.6 (35.0)	<.001
≥65	18 396	37 (2.1)	229 (2.2)	.77	355 (14.0)	2627 (20.5)	<.001	11.7 (9.4)	9.6 (6.4)	<.001	76.2 (55.9)	53.1 (35.3)	<.001
**GI hemorrhage**													
<65	15 009	87 (2.5)	218 (3.3)	.02	700 (14.4)	1466 (19.1)	<.001	3.8 (5.0)	4.0 (4.4)	.01	14.4 (23.6)	11.9 (17.0)	<.001
≥65	65 174	288 (4.1)	2119 (5.8)	<.001	1608 (17.3)	8275 (18.5)	.006	4.4 (5.1)	4.3 (3.8)	.009	16.3 (19.8)	11.3 (13.1)	<.001
**HF**													
<65	26 730	153 (2.3)	323 (3.0)	.004	1993 (20.9)	3322 (26.6)	<.001	5.2 (5.7)	5.1 (6.0)	.05	17.0 (21.7)	15.0 (31.2)	<.001
≥65	156 863	1235 (6.0)	8742 (10.4)	<.001	6009 (22.3)	25 672 (24.6)	<.001	5.4 (5.7)	4.9 (4.6)	<.001	16.9 (22.5)	11.6 (19.0)	<.001
**Pneumonia**													
<65	19 476	186 (3.3)	334 (3.9)	.07	1083 (15.4)	1894 (18.9)	<.001	5.0 (6.7)	4.8 (4.9)	.01	17.6 (33.0)	11.1 (16.3)	<.001
≥65	105 275	965 (7.2)	5785 (9.7)	<.001	2817 (16.5)	13 934 (19.3)	<.001	5.4 (6.7)	5.0 (4.5)	<.001	17.8 (28.4)	10.2 (12.3)	<.001
**Stroke**													
<65	16 223	53 (2.2)	541 (6.3)	<.001	465 (13.7)	2764 (27.5)	<.001	5.3 (8.3)	6.3 (9.5)	<.001	17.7 (28.2)	21.3 (34.5)	<.001
≥65	67 812	331 (7.0)	6494 (16.5)	<.001	1000 (15.5)	14 512 (30.0)	<.001	5.8 (7.3)	5.0 (5.5)	<.001	19.0 (28.3)	15.6 (20.6)	<.001

Abbreviations: AMI, acute myocardial infarction; CABG, coronary artery bypass surgery; GI, gastrointestinal; HF, heart failure; LOS, length of stay; VA, Veterans Affairs.

^a^ Nos. varied by outcome and reported only for LOS.

^b^ *P* values reported for analysis of variance tests.

Younger VA patients had higher probability of readmission for AMI than non-VA patients (mean [SD], 0.21 [0.41] vs 0.19 [0.39]; *P* = .004). VA hospitalizations had longer mean (SD) LOS than non-VA hospitalizations for all conditions mostly among older patients (age ≥65 years, AMI, 5.2 [6.4] vs 4.7 [5.0]; CABG, 11.7 [9.4] vs 9.6 [6.4]; GI hemorrhage, 4.4 [5.1] vs 4.3 [3.8]; HF, 5.4 [5.7] vs 4.9 [4.6]; pneumonia, 5.4 [6.7] vs 5.0 [4.5]; stroke, 5.8 [7.3] vs 5.0 [5.5], respectively. Mean (SD) inpatient costs were mostly higher in VA hospitalizations (AMI: age ≥65 years, $24 600 [$32 000] vs $21 700 [$24 800]; *P* <.001; CABG: age <65 years, $68 500 [$52 800] vs $51 600 [$35 000]; *P* <.001; age ≥65 years, $76 200 [$55 900] vs $53 100 [$35 300]; *P* <.001; GI hemorrhage: age <65 years, $14 400 [$23 600] vs $11 900 [$17 000]; *P* <.001; age ≥65 years, $16 300 [$19 800] vs $11 300 [$13 100]; *P* <.001; HF: age <65 years, $17 000 [$21 700] vs $15 000 [$31 200]; *P* <.001; age ≥65 years, $16 900 [$22 500] vs $11 600 [$19 000]; *P* <.001; pneumonia: age <65 years, $17 600 [$33 000] vs $11 100 [$16 300]; *P* <.001; age ≥65 years, $17 800 [$28 400] vs $10 200 [$12 300]; *P* <.001; stroke: age ≥65 years, $19 000 [$28 300] vs $15 600 [$20 600]; *P* <.001) except for younger patients with AMI and stroke who had higher costs in non-VA hospitalizations ($23 800 [$24 900] vs $22 500 [$22 500]; *P* = 005; and $21 300 [$34 500] vs $17 700 [$28 200]; *P* <.001).

### Average Treatment Outcomes of VA Hospitals

In models balancing covariates between patients in VA and non-VA hospitals, there were no significant treatment effects of VA hospitals on probability of 30-day mortality for most conditions (Table 3). VA hospitalizations had significantly lower probability of mortality for HF for veterans aged 65 and older (−0.02 [95% CI,−0.03 to −0.01]) and stroke for both age groups (age <65 years, −0.03 [95% CI, −0.05 to −0.02]; age ≥65 years, −0.05 [95% CI, −0.07 to −0.03]).

**Table 3.** Average Treatment Outcomes of VA Hospitals Compared With Non-VA Hospitals for 30-Day Mortality and 30-Day Readmission

Condition and age group, years	30-d Mortality^a^			30-d Readmission		
	Participants mortality, No.	Average treatment outcome (95% CI)	*P* value	Participants readmission, No.	Average treatment outcome (95% CI)	*P* value
**AMI**						
<65^b^	15 105	−0.007 (−0.016 to 0.003)	.17	17 627	0.037 (0.014 to 0.060)	.002
≥65	47 288	0.012 (−0.009 to 0.033)	.26	57 713	0.001 (−0.022 to 0.025)	.90
**CABG**						
<65	3795	Not estimable		4510	−0.035 (−0.060 to −0.011)	.00
≥65	12 037	0.009 (−0.004 to 0.021)	.17	14 519	−0.045 (−0.074 to −0.017)	.001
**GI hemorrhage**						
<65	9511	−0.001 (−0.010 to 0.008)	.80	10 977	−0.043 (−0.060 to −0.026)	<.001
≥65	42 329	0.004 (−0.009 to 0.016)	.58	51 142	−0.0001 (−0.019 to 0.021)	.91
**HF**						
<65	16 295	−0.003 (−0.009 to 0.004)	.41	18 883	−0.049 (−0.066 to −0.032)	<.001
≥65	101 388	−0.017 (−0.027 to −0.006)	.001	123 584	−0.008 (−0.024 to 0.008)	.31
**Pneumonia**						
<65	13 162	−0.001 (−0.008 to 0.006)	.76	15 355	−0.042 (−0.056 to −0.028)	<.001
≥65	70 658	−0.004 (−0.015 to 0.008)	0.54	84 705	−0.029 (−0.043 to −0.015)	<.001
**Stroke**						
<65	10 537	−0.033 (−0.045 to −0.022)	<.001	12 288	−0.109 (−0.132 to −0.086)	<.001
≥65	43 052	−0.053 (−0.074 to −0.031)	<.001	52 555	−0.130 (−0.158 to −0.101	<.001

Abbreviations: AMI, acute myocardial infarction; CABG, coronary artery bypass surgery; GI, gastrointestinal; HF, heart failure; VA, Veterans Affairs.

^a^ Average treatment outcomes of difference in predicted probability for VA hospitals vs non-VA hospitals estimated from inverse probability weighting regression adjustment models with probit models for treatment and outcomes.

^b^ Nos. varied by outcome.

VA hospitalizations for AMI had higher probability of 30-day readmission only among younger veterans (0.04 [95% CI, 0.01 to 0.06]). VA hospitalizations had significantly lower probability of 30-day readmission for CABG (age <65 years, −0.04 [95% CI, −0.06 to −0.01]; age ≥65 years, −0.05 [95% CI, −0.07 to −0.02]), GI hemorrhage (age <65 years, −0.04 [95% CI, −0.06 to −0.03]), HF (age <65 years, −0.05 [95% CI, −0.07 to −0.03]), pneumonia (age <65 years, −0.04 [95% CI, −0.06 to −0.03]; age ≥65 years, −0.03 [95% CI, −0.04 to −0.02]), and stroke (age <65 years, −0.11 [95% CI, −0.13 to −0.09]; age ≥65 years, −0.13 [95% CI, −0.16 to −0.10]).

There was significantly greater mean LOS in VA hospitals for all study conditions and both age groups except stroke in younger patients (Table 4). Differences in LOS between VA and non-VA hospitals ranged from 0.28 (95% CI, 0.09 to 0.47) days for GI hemorrhage among younger patients to 3.00 (95% CI, 2.43 to 3.57) days for CABG among older patients. Mean costs (log transformed) of VA hospitalizations for AMI among younger veterans were approximately 7% lower than non-VA hospitalizations (age <65 years, −0.07 [95% CI, −0.11 to −0.02]) but 21% higher among older veterans (age ≥65 years, 0.21 [95% CI, 0.17 to 0.25]). Mean hospitalization costs were significantly higher in VA hospitals for other study conditions and age groups, except for stroke among younger patients. Full results are in eTables 1 to 12 in Supplement 2.

**Table 4.** Average Treatment Outcomes of VA Hospitals Compared With Non-VA Hospitals for Length of Stay and Costs

Condition and age group, years	LOS, d		*P* value	Costs in $ (log transformed)		*P* value
	Participants LOS, No.	Average treatment outcome, (95% CI)^a^		Participants costs, No.	Average treatment outcome, (95% CI)^a^	
**AMI**						
<65^b^	22 681	0.97 (0.50 to 1.4)	<.001	22 057	−0.07 (−0.11 to −0.02)	.003
≥65	74 138	1.41 (1.09 to 1.7)	<.001	71 609	0.21 (0.17 to 0.25)	<.001
**CABG**						
<65	5829	2.31 (1.77 to 2.84)	<.001	5734	0.32 (0.28 to 0.35)	<.001
≥65	18 396	3.00 (2.43 to 3.57)	<.001	18 030	0.39 (0.35 to 0.44)	<.001
**GI hemorrhage**						
<65	15 009	0.28 (0.09 to 0.47)	.003	14 517	0.24 (0.21 to 0.27)	<.001
≥65	65 174	0.50 (0.30 to 0.70)	<.001	62 712	0.40 (0.36 to 0.44)	<.001
**HF**						
<65	26 730	1.22 (0.99 to 1.45)	<.001	25 956	0.38 (0.35 to 0.41)	<.001
≥65	156 863	1.29 (1.12 to 1.46)	<.001	150 851	0.50 (0.48 to 0.53)	<.001
**Pneumonia**						
<65	19 476	0.53 (0.32 to 0.73)	<.001	18 775	0.36 (0.33 to 0.39)	<.001
≥65	105 275	0.57 (0.41 to 0.74)	<.001	99 469	0.47 (0.45 to 0.50)	<.001
**Stroke**						
<65	16 223	0.88 (−0.13 to 1.89)	.09	15 643	0.04 (−0.01 to 0.09)	.10
≥65	67 812	2.34 (1.58 to 3.10)	<.001	65 045	0.40 (0.33 to 0.48)	<.001

Abbreviations: AMI, acute myocardial infarction; CABG, coronary artery bypass surgery; GI, gastrointestinal; HF, heart failure; LOS, length of stay; VA, Veterans Affairs.

^a^ Average treatment outcomes of difference in predicted probability for VA hospitals vs non-VA hospitals estimated from inverse probability weighting regression adjustment models with probit models for treatment and linear models for outcomes.

^b^ Nos. varied by outcome.

In sensitivity analyses with all states, VA hospitals had significantly higher probability of in-hospital mortality for pneumonia but significantly lower probability for stroke and no other differences (eTable 19 in Supplement 2). Including only nonelective admissions, results were similar to all hospitalizations (eTable 20 in Supplement 2). When analysis was limited to only 1 observation per patient, results were similar to all hospitalizations, except that mortality was higher in VA hospitals for AMI for patients younger than 65 years (eTable 21 in Supplement 2).

## Discussion

To our knowledge, this is the first study to compare outcomes for veterans of all ages in VA and non-VA hospitals for 6 common conditions. After accounting for selection of patients into VA or non-VA hospitals, patients treated in VA hospitals had significantly lower probability of 30-day mortality than those in non-VA hospitals for HF among older patients and stroke for both younger and older patients. Patients treated for CABG, GI hemorrhage, HF, pneumonia, and stroke in VA hospitals had lower probability of readmission compared with patients in non-VA hospitals; however, differences for GI hemorrhage and HF were found only in younger patients. In contrast, younger patients hospitalized for AMI in VA hospitals had higher probability of readmission than non-VA patients. Mean hospitalization costs were mostly higher, and mean LOS was longer in VA hospitals for the study conditions. Costs of AMI hospitalizations for younger patients were lower in VA hospitals than non-VA hospitals.

Our findings showing lower mortality in VA hospitals for 2 of the 6 conditions suggests that there was a mortality advantage associated with VA hospitals but not for all types of care. Recent studies of inpatient surgery and emergency department care also found associations between lower mortality and better quality in VA hospitals compared with non-VA hospitals.^5,46,47^ More research is needed to determine what aspects of VA care, such as postdischarge care, can improve mortality and whether there are differences for other clinical outcomes.

Our findings on mortality are similar to some previous findings but diverged from others. We did not find differences in mortality for CABG between VA and non-VA hospitals similar to another study.^17^ We found lower mortality for HF and stroke but not AMI in VA hospitals, while another study found lower mortality for AMI and HF in VA hospitals but did not include stroke in a study of adults aged 65 years and older.^13^ That study included veterans and nonveterans in an earlier period, which may explain different findings.^8^

We found lower readmissions in VA hospitals for CABG, GI hemorrhage, HF, pneumonia, and stroke but higher readmissions for AMI in younger patients. In contrast, Nuti et al^8^ documented higher readmissions in VA hospitals for AMI, HF, and pneumonia in older patients prior to access expansions. VA hospitals may be more successful in reducing readmissions due to an integrated delivery system, implementation of the patient-centered medical home, and an electronic medical record system. It is unclear why younger patients who were hospitalized for AMI were more likely to be readmitted in VA hospitals even though patients often travel longer distances to VA hospitals, potentially affecting their outcomes. Both VA and non-VA hospitals have recently emphasized reducing readmissions through the use of performance measures in the VA and payment policies in the private sector and Medicare.

Mean LOS was longer and costs were higher in VA hospitalizations for most conditions compared with non-VA hospitalizations. Medicare and private insurance payment policies (eg, bundled payment programs) have focused on efficiency and may have influenced hospitals to discharge patients sooner while VA hospitals were unaffected by such policies. VA hospitals may keep patients longer to ensure they are stable before discharging them. Higher VA hospitalization costs may be partly explained by longer LOS. There may be other differences due to staffing and overhead between VA and non-VA hospitals leading to greater resource use. A study^48^ about ED care found lower VA costs over 28 days, so focusing on inpatient costs does not account for post-discharge costs that may be lower in the VA.

Our findings are especially relevant given that the Centers for Medicare & Medicaid Services now publicly reports the performance of VA hospitals in addition to non-VA hospitals on its Care Compare website. Veterans may be more likely to choose VA hospitals that perform comparatively better than other hospitals in their service area.

### Limitations

This study has limitations. These data precede the MISSION Act of 2018, so our findings may not be generalizable to veterans currently accessing non-VA care. Our findings were based on hospitalizations from 47% of VA hospitals from diverse states, but they may not be generalizable to all VA hospitals. Our methods used many observed patient characteristics to account for patient selection, but there may have been unobserved factors which influenced patients’ use of VA hospitals and outcomes. Undercoding of comorbidities was previously documented in the VA,^49,50^ so differences in outcomes may have been underestimated. We did not distinguish between potentially avoidable readmissions and unavoidable or planned readmissions which may have led to overestimates of the observed readmission rates; however, planned readmissions only account for roughly 7% of all readmissions, so it is unlikely to materially affect our results.^51^ Non-VA hospitalization costs were estimated from cost-adjusted charges, which is less accurate than production costs, so cost differences may have been underestimated. Finally, we included hospitalizations for patients who were discharged against medical advice because these hospitalizations typically represent a small proportion (1%) of hospitalizations.^52^

## Conclusions

Expanding access to non-VA care may improve timeliness and reduce travel costs for many veterans; however, there are tradeoffs with higher mortality and readmissions in non-VA hospitals observed across age groups. As more veterans use care in the community paid for by the VA due to the MISSION Act, our findings suggest there may be reasons for concern. Veterans could experience worse outcomes for some types of care without well-developed community care networks based on quality standards and sufficient care coordination between VA and non-VA clinicians. In an era of greater choice, veterans’ often benefit by choosing VA care.

## Notes

Supplement 1.
eMethods.

eReferences.

Supplement 2. **eTable 1.** AMI Patients Less Than 65 Years of Age: Coefficients From IPWRA Models **eTable 2.** AMI Patients 65 Years and Older: Coefficients From IPWRA Models **eTable 3.** CABG Patients Less Than 65 Years of Age: Coefficients From IPWRA Models **eTable 4. **CABG Patients 65 Years and Older: Coefficients From IPWRA Models **eTable 5. **GI Hemorrhage Patients Less Than 65 Years of Age: Coefficients From IPWRA Models **eTable 6.** GI Hemorrhage Patients 65 Years and Older: Coefficients From IPWRA Models **eTable 7.** HF Patients Less Than 65 Years of Age: Coefficients From IPWRA Models **eTable 8.** HF Patients 65 Years and Older: Coefficients From IPWRA Models **eTable 9.** Pneumonia Patients Less Than 65 Years of Age: Coefficients From IPWRA Models **eTable 10.** Pneumonia Patients 65 Years and Older: Coefficients From IPWRA Models **eTable 11.** Stroke Patients Less Than 65 Years of Age: Coefficients From IPWRA Models **eTable 12.** Stroke Patients 65 Years and Older: Coefficients From IPWRA Models **eTable 13.** Balance of Covariates in Mortality Model for AMI **eTable 14. **Balance of Covariates in Mortality Model for CABG **eTable 15.** Balance of Covariates in Mortality Model for GI Hemorrhage **eTable 16.** Balance of Covariates in Mortality Model for HF **eTable 17.** Balance of Covariates in Mortality Model for Pneumonia **eTable 18.** Balance of Covariates in Mortality Model for Stroke **eTable 19.** In-Hospital Mortality: Average Treatment Effects (ATE) and Predicted Probability/Means for VA Hospitals From IPWRA Models **eTable 20.** Results From IPWRA Models Including Only Nonelective Hospitalizations **eTable 21.** Results From IPWRA Models With One Observation per Patient and Standard Errors Adjusted for Clustering Within Hospital **eTable 22.** *ICD-9* and *ICD-10* Codes for Admitting Condition and Comorbidities

Supplement 3.
Data Sharing Statement
