# Supplementary material for: Outcomes of Veterans Treated in Veterans Affairs Hospitals vs Non–Veterans Affairs Hospitals
Source: JAMA Netw Open. 2023 Dec 1;6(12):e2345898. doi: 10.1001/jamanetworkopen.2023.45898 (PMC10692833; doi:10.1001/jamanetworkopen.2023.45898)
Supplement: Supplement 1. — eMethods. eReferences. [file jamanetwopen-e2345898-s001.pdf]

Supplemental Online Content

Yoon J, Phibbs CS, Ong MK, et al. Outcomes of veterans treated in Veterans Affairs hospitals vs non-Veterans Affairs hospitals. *JAMA Netw Open*. 2023;6(12):e2345898. doi:10.1001/jamanetworkopen.2023.45898

eMethods.

eReferences.

This supplemental material has been provided by the authors to give readers additional information about their work.

© 2023 Yoon J et al. *JAMA Netw Open.*

1. eMethods Details

Cohort and data sources

Veterans included in our enrollee cohort had to be enrolled in the VA health care system as recorded in the VA Assistant Deputy Under Secretary for Health (ADUSH) Enrollment File during the study years (calendar years 2012-2017) with an address in one of the 11 study states (AZ, CA, CT, FL, IL, LA, MA, MO, NY, PA, SC) and complete information for age, gender, and valid name or social security number. There was a total of 21.6 million Veteran-years that met these criteria and were eligible for data linkage with state inpatient discharge data.

VA hospitalization data

All VA data were obtained from the VA Corporate Data Warehouse (CDW). VA hospitalization records were obtained from VA CDW Inpatient data. VA Inpatient data included the bed section or location of care, and we restricted records to those with bed sections for medicine and surgery. Patients could be transferred during their hospitalization from a medicine or surgery bed section to another bed section (e.g. rehabilitation), but we measured only the length of stay, costs, diagnoses, and outcomes that occurred in medicine and surgery bed sections. VA hospitalization costs were obtained from the VA Managerial Cost Accounting (MCA) System data which reports the cost of all VA inpatient and outpatient care. MCA costs are based on a bottom-up costing method in which relative values are assigned to health care encounters, and the resources involved in producing encounters are used to estimate the production costs of care.[1] VA costs of care include direct, indirect, and central administrative costs to operate the VA health care system nationally; we removed a portion of indirect costs previously estimated to be spent on research, teaching, and national administration to obtain costs of care more comparable to non-VA hospitals.[2]

Non-VA hospitalization data

We reviewed availability and policies to obtain state inpatient discharge data for research for all 50 states to determine the feasibility of obtaining data for Veterans enrolled in the VA using linkage of personal identifiers. Ultimately, non-VA hospitalization records were obtained from 11 state agencies that manage research requests for state hospital discharge data (Arizona Department of Health Services, California Department of Health Care Access and Information, Connecticut Department of Public Health, Florida Agency for Health Care Administration, Illinois Department of Public Health, Louisiana Department of Health, Massachusetts Center for Health Information and Analysis, Missouri Department of Health and Senior Services, New York Statewide Planning and Research Cooperative System, Pennsylvania Health Care Cost Containment Council, and South Carolina Revenue and Fiscal Affairs Office). States conducted a data linkage using VA patient identifiers such as name, social security number, date of birth, sex, and race/ethnicity to link data on VA enrollees in each state to discharge records using probabilistic or deterministic methods.[3,4] Discharge data were received under research requests to state agencies; each agency conducted a data linkage using a combination of personal identifiers based on state guidelines, state institutional review board approval, and data use agreements.

Since discharge data from 2012 could not be obtained from IL, all hospitalization records from IL were excluded for that year in our analysis. Non-VA hospitalization costs were estimated based on the total charges reported for each hospitalization adjusted by a hospital-specific cost-to-charge ratio obtained from hospitals' CMS cost reports.[5] There were missing or $0 charges reported for 23,258 non-VA hospitalizations, so these records were excluded from analysis on costs. Both VA and non-VA costs were adjusted for inflation to 2017 dollars based on the GDP deflator.[6]

In our analysis we excluded hospitalizations that had a length of stay longer than 180 days since these were not considered acute hospitalizations,[7] and hospitalizations that occurred within 30 days of the previous admission for a patient in order to measure outcomes of the index hospitalization. We excluded transfers to another hospital and observation stays.

Dependent variables

Thirty-day mortality was indicated if the date of death, reported in the VA Vital Status file, occurred within 30 days of the date of admission. 30-day readmission was indicated for all-causes and included both planned and unplanned readmissions; only 7% of readmissions are estimated to be planned.[8] Readmission was indicated regardless of where the stays occurred, so a patient could have an index stay in VA hospital and a readmission in a

non-VA hospital and vice versa. Length of stay was measured in number of days, and total costs of hospitalizations were measured in dollars in the analysis.

Independent variables

In inverse probability weighted regression adjustment models (IPWRA), we estimated a treatment equation and an outcome equation and included independent variables for patient characteristics previously identified as being related to use of VA care in the treatment equation and those related to hospital outcomes in the outcomes equation.[7, 9, 10] Veterans' sociodemographic characteristics were obtained from VA Observational Medical Outcomes Partnership (OMOP) Files and community characteristics obtained U.S. Census data. We used a continuous measure of age, gender was categorized as male or female, marital status was categorized as married, divorced/separated/widowed, single, or unknown marital status, and VA enrollment category was grouped by VA-assigned priority for care based on service-connected disabilities and incomes below the VA means test from highest priority (groups 1-2) to lowest priority (groups 7-8).

We used Veterans' race/ethnicity from VA data if it was available; it was obtained from the electronic health record, and categories included Black (Hispanic or Non-Hispanic), Hispanic, Non-Hispanic White, and Other (including Asian American, Pacific Islanders, American Indian, and Alaska Native). For patients who had missing race/ethnicity in VA data, we used race/ethnicity as reported in state discharge data. Veterans with unknown race/ethnicity (6%) were included in the cohort since it was not the primary independent variable.

Patients' distance to their hospital was measured based on the straight-line distance from their residence to the hospital location. Veterans' rurality was categorized as urban or rural based on Rural-Urban Continuum Codes (RUCA) developed by the U.S. Department of Agriculture that distinguishes counties by population density and adjacency to metro areas and degrees of urbanization. U.S. Census data for median income and unemployment rate was linked to Veterans' zip codes. State of residence was grouped into regions for Northeast (NY, MA, PA, CT), South (SC, LA, FL), Midwest (IL, MO), and West (CA, AZ) and included as dummy variables with West as the reference group.

We also measured patients' comorbidity for each stay using the Elixhauser-van Walvraven index calculated from the diagnosis related group (DRG) and all recorded diagnosis codes.[11] We also included separate indicators for comorbid medical conditions that were used to calculate the Elixhauser-van Walraven score: heart failure, cardiac arrythmias, valvular disease, pulmonary circulation disease, peripheral vascular disease, neurological disorders, chronic pulmonary disease, diabetes with complications, diabetes without complications, hypertension, hypothyroidism, arthritis, renal failure, liver disease, coagulopathy, lymphoma, metastatic cancer, solid tumor without metastasis, rheumatoid arthritis, obesity, fluid and electrolyte disorders, weight loss, chronic blood loss anemia, deficiency anemias. Mental health comorbidity was indicated for mood disorders (including depression), substance use disorders, serious mental illness, posttraumatic disorder, and personality disorders.

Admission type of non-elective (e.g. emergency, urgent, trauma), elective, or other care for non-VA stays was obtained directly from discharge records as reported on billing forms. MO records only contained admission type for 2016-2017, so earlier years were included in analysis as other admission type. VA inpatient data do not report an admission type, so it was assigned based on the admission type assigned to the diagnosis related group (DRG) and primary diagnosis of non-VA hospitalizations.

Independent variables for Elixhauser-van Walraven index, admission type, and medical and mental health comorbidities varied for each hospitalization. Variables for patients' age, rurality, state of residence, and Census information varied for each year. Patients' gender, race/ethnicity, marital status, and VA priority group was assigned based on the most recent year and was fixed over the study period.

Analytic methods

The unit of analysis was the hospital stay. From the original data limited to index hospital stays for the six study conditions (N=616,917), we excluded hospitalization records of patients with missing data for age (N=21), comorbidity information (N=5), residential zip code (N=2,056), marital status (N=6,187), or Census information (N=14,350) for a final sample size of 593,578. Observations that had missing or $0 costs (N=23,258) were dropped from analysis on costs. Non-VA hospital stays in CA and PA did not contain specific admission date or discharge dates, and non-VA stays in CA did not contain a readmission indicator, so observations from CA were dropped from analysis of 30-day readmission (N=104,187), and observations from CA and PA were dropped from analysis of 30-day mortality (N=198,956) since these measures could not be calculated.

IPWRA is a doubly robust method using propensity scores to reweight the outcome of observations by their inverse probability of treatment. It combines inverse probability treatment weighting with regression adjustment. The treatment model (VA versus non-VA hospital) is estimated first, and then the predicted probability of treatment is used to compute inverse-probability weights that are applied in the outcomes equation to create weighted

© 2023 Yoon J et al. *JAMA Netw Open.*

observations with balanced covariates. Observations that have higher likelihood of treatment are up-weighted, and those that have lower likelihood are down-weighted. Only one of the treatment or outcomes equation needs to be correctly specified to obtain unbiased estimates of the average treatment effect.

Treatment models predicting use of VA hospital relative to non-VA hospital were developed using all measured covariates potentially related to selection of VA hospitals and comparing model diagnostics for larger and more parsimonious models including area under the receiver operating characteristic (ROC) curve and the Hosmer-Lemeshow chi-square goodness-of-fit test.[12] In final treatment models, area under the ROC curve varied by condition from 0.74 for CABG to 0.80 for pneumonia. Treatment models for AMI, HF, and pneumonia included age, gender, marital status, race/ethnicity, VA priority status, overall comorbidity score, substance use disorder comorbidity, posttraumatic stress disorder comorbidity, distance to closest VA hospital (categorized by quartile), geographic region, rurality, area-level income, and post-Choice Act period. Treatment models for CAGB, GI hemorrhage, and stroke included many of the same covariates as the other conditions but excluded variables including gender, race/ethnicity, rurality, and area-level income since these factors were not related to treatment. Treatment equations predicting use of VA or non-VA hospital used a probit model.

Similarly, we compared outcomes models with larger and more parsimonious models using model diagnostics to refine our final models. In final mortality models, area under the ROC curve varied by condition from 0.72 for HF to 0.77 for GI hemorrhage. Final outcome models included age, marital status, race/ethnicity, VA priority status, non-elective admission, overall comorbidity score, separate indicators for medical comorbidities, any mental health comorbidity, and area-level income. Not all medical comorbidity indicators could be included when estimating mortality and readmission for all six study conditions due to low rates of some indicators without variation in the outcome for certain conditions. The outcome equations used probit models for mortality and readmissions and linear regression models for LOS and the log-transformed costs. Standard errors were adjusted for clustering within each unique patient-hospital combination since some patients had more than one hospitalization, including in more than one hospital and more than one study condition, during the study period. Full regression estimates are reported in eTables 1-12. IPWRA analyses were performed in StataMP 18 use teffects. We followed the STROBE guidelines for reporting cohort studies (checklist included below).

We assessed balance of covariates for mortality models, and standardized differences of covariates in weighted samples for all conditions were less than -0.08 except for age (standardized difference for age in GI model=-0.13) with variance ratios of covariates very close to 1.0 with the exception of Elixhauser score for CABG models and area-level income for HF (variance ratio =0.79 and 1.21, respectively). Balance characteristics are reported below in eTables 13-18.

Sensitivity analyses

Since we could not include hospitalizations in CA and PA in our models for 30-day mortality, we used in-hospital mortality as a secondary outcome (eTable 19). We estimated in-hospital mortality including data from all states in IPWRA models with all ages included. We also limited hospitalizations to non-elective admissions (78% of all hospitalizations) since non-elective and elective admissions may have different patterns in treatment and outcomes between hospitals (eTable 20).

We also conducted analysis limiting the sample to one hospitalization per patient since a small minority of patients (30%) had more than one hospitalization due to having hospitalizations for more than one study condition or multiple hospitalizations for the same condition over the six-year period. Standard errors were adjusted for clustering within hospitals (eTable 21).

Hospital characteristics

For descriptive purposes, we measured hospital characteristics. For all hospitals we obtained hospital characteristics that included the bed occupancy rate which was calculated as the mean number of beds occupied divided by the mean total number of staffed beds in each year. Academic affiliation for non-VA hospitals was measured from the CMS Provider of Service Hospital/Non-Hospital Facilities file as those with a major affiliation to a medical school.[13] For VA hospitals we indicated which facilities had a strong academic affiliation as measured by facilities with large (and medium-large) research and teaching programs. Patient experience for both VA and non-VA hospitals was obtained from the Consumer Assessment of Health Providers and Systems measure for percent of patients recommending their hospital to others.[14]

© 2023 Yoon J et al. *JAMA Netw Open.*

Diagnosis codes

All ICD-9 and ICD-10 diagnosis codes used to identify the study conditions and comorbidities are listed in eTable 22.

© 2023 Yoon J et al. *JAMA Netw Open.*

eReferences

1. Chapko MK, Liu CF, Perkins M, Li YF, Fortney JC, Maciejewski ML. Equivalence of two healthcare costing methods: bottom-up and top-down. *Health economics*. 2009;18(10):1188-1201.
2. Barnett PG, Berger M. *Indirect Costs of Specialized VA Inpatient Mental Health Treatment*. 2003. *Technical Report #6*.
3. Li B, Quan H, Fong A, Lu M. Assessing record linkage between health care and Vital Statistics databases using deterministic methods. *BMC health services research*. 2006;6:1-10.
4. Méray N, Reitsma JB, Ravelli AC, Bonsel GJ. Probabilistic record linkage is a valid and transparent tool to combine databases without a patient identification number. *Journal of clinical epidemiology*. 2007;60(9):883. e1-883. e11.
5. Cutting AC, Goldberg GM, Jervis KJ. An easier method to extract data from large databases: The Medicare hospital cost report database. *Research in Healthcare Financial Management*. 2007;11(1):27.
6. Bureau of Economic Analysis. GDP Price Deflator. US Department of Commerce; 2017.
7. Carey K, Lin M-Y. Hospital length of stay and readmission: an early investigation. *Medical Care Research and Review*. 2014;71(1):99-111.
8. Horwitz LI, Grady JN, Cohen DB, et al. Development and validation of an algorithm to identify planned readmissions from claims data. *Journal of hospital medicine*. 2015;10(10):670-677.
9. Allison JJ, Kiefe CI, Weissman NW, et al. Relationship of hospital teaching status with quality of care and mortality for Medicare patients with acute MI. *Jama*. 2000;284(10):1256-1262.
10. Silva GC, Jiang L, Gutman R, et al. Mortality Trends for Veterans Hospitalized With Heart Failure and Pneumonia Using Claims-Based vs Clinical Risk-Adjustment Variables. *JAMA Internal Medicine*. 2020-03-01 2020;180(3):347. doi:10.1001/jamainternmed.2019.5970
11. van Walraven C, Austin PC, Jennings A, Quan H, Forster AJ. A modification of the Elixhauser comorbidity measures into a point system for hospital death using administrative data. *Medical care*. 2009:626-633.
12. Hosmer DW, Hosmer T, Le Cessie S, Lemeshow S. A comparison of goodness-of-fit tests for the logistic regression model. *Statistics in medicine*. 1997;16(9):965-980.
13. Schrag D, Bach PB, Dahlman C, Warren JL. Identifying and measuring hospital characteristics using the SEER-Medicare data and other claims-based sources. *Medical care*. 2002:IV96-IV103.
14. Goldstein E, Farquhar M, Crofton C, Darby C, Garfinkel S. Measuring hospital care from the patients' perspective: An overview of the CAHPS® hospital survey development process. *Health services research*. 2005;40(6p2):1977-1995.
