# Supplementary material for: Outcomes of Veterans Treated in Veterans Affairs Hospitals vs Non–Veterans Affairs Hospitals
Source: JAMA Netw Open. 2023 Dec 1;6(12):e2345898. doi: 10.1001/jamanetworkopen.2023.45898 (PMC10692833; doi:10.1001/jamanetworkopen.2023.45898)
Supplement: Supplement 3. — Data Sharing Statement [file jamanetwopen-e2345898-s003.pdf]

Data Sharing Statement

Data
Data available: No

Additional Information
Explanation for why data not available: Data are unable to be shared since data from state public health agencies were obtained through a memorandum of understanding that limits data access to the study team.
